# Serum Histatin Levels in COVID‐19: Association With Disease Severity and Immune Status

**DOI:** 10.1002/jmv.71009

**Published:** 2026-06-10

**Authors:** Umut Aydın, Deccane Düzenci, Ramazan Fazıl Akkoç, Ahmet Karataş

**Affiliations:** ^1^ Department of Medical Oncology, Faculty of Medicine İnönü University Malatya Turkey; ^2^ Department of Intensive Care Fethi Sekin City Hospital Elazığ Turkey; ^3^ Department of Anatomy, Faculty of Medicine Fırat University Elazığ Turkey; ^4^ Department of Rheumatology, Faculty of Medicine Fırat University Elazığ Turkey

**Keywords:** antimicrobial peptides, biomarkers, corticosteroids, COVID‐19 severity, histatins, inflammation, SARS‐CoV‐2

## Abstract

Histatins are histidine‐rich antimicrobial peptides with emerging immunomodulatory properties; however, their systemic behavior across the clinical spectrum of COVID‐19 remains unknown. We aimed to characterize serum histatin levels in patients with mild COVID‐19 (without pneumonia) and severe COVID‐19 (with pneumonia) and to explore factors potentially associated with their variation. Ninety individuals were prospectively enrolled: healthy controls (*n* = 30), patients with mild COVID‐19 (without pneumonia (*n* = 30), and patients with severe COVID‐19 (with pneumonia receiving corticosteroid therapy (*n* = 30). Serum histatin‐1, histatin‐3, and histatin‐5 concentrations were quantified by ELISA. Group comparisons were performed using Kruskal–Wallis and Mann–Whitney *U* tests; correlations were assessed using Pearson coefficients. Histatin‐1 did not differ among groups (*p* = 0.161). Both histatin‐3 and histatin‐5 were significantly reduced in mild COVID‐19 compared with controls (*p* = 0.004 and *p* = 0.002, respectively). Unexpectedly, patients with severe COVID‐19, all of whom were receiving corticosteroids, did not exhibit further histatin reductions; their levels were comparable to controls and significantly higher than the mild group (histatin‐3 *p* = 0.016; histatin‐5 *p* = 0.028). Histatin‐3 correlated significantly with ferritin (*r* = 0.413; *p* = 0.009), D‐dimer (*r* = 0.369; *p* = 0.021), and albumin (*r* = −0.330; *p* = 0.018). SARS‐CoV‐2 infection without pneumonia was associated with significantly lower serum histatin‐3 and histatin‐5 levels. In contrast, histatin levels were preserved in patients with severe COVID‐19 and pneumonia who were receiving corticosteroid therapy. Because of the cross‐sectional design, this finding should be interpreted as an association rather than evidence of a treatment effect. The observed correlations between histatin‐3 and ferritin, D‐dimer, and albumin further suggest that histatin‐3 may be linked to acute‐phase and inflammatory responses during COVID‐19.

## 1 Introduction

More than 5 years after the emergence of SARS‐CoV‐2, the identification of reliable biomarkers that reflect the immune status of patients across the clinical spectrum of COVID‐19 remains a priority. Conventional inflammatory markers such as C‐reactive protein and ferritin, while valuable, do not fully capture the complex immunological derangements underlying disease progression [1, 2]. This has stimulated interest in antimicrobial peptides, which occupy a unique position at the interface of innate immunity and inflammatory regulation [3].

Histatins are a family of small, histidine‐rich cationic peptides, principally histatin‐1, histatin‐3, and histatin‐5, predominantly secreted by the parotid and submandibular salivary glands [4]. Beyond their well‐characterized antimicrobial functions, histatins possess immunomodulatory properties of potential relevance to viral infections: histatin‐1 attenuates lipopolysaccharide‐induced inflammatory signaling [5], histatin‐3 modulates Toll‐like receptor pathways [6], and histatin‐5 enhances cellular defenses against oxidative stress [7].

SARS‐CoV‐2 has been shown to directly infect salivary gland epithelium via ACE2‐mediated entry, raising the possibility that infection may disrupt histatin production at the source [8]. Previous studies have documented alterations in salivary composition among patients with COVID‐19 [9, 10]; however, whether circulating histatin levels are systematically altered across different severities of COVID‐19, and whether such alterations differ between patients with and without pulmonary involvement, remains unexplored.

The primary objective of this study was to characterize serum histatin‐1, histatin‐3, and histatin‐5 levels in patients with mild COVID‐19 (without pneumonia) and severe COVID‐19 (with pneumonia), compared with healthy controls. A secondary objective was to explore factors potentially associated with variation in histatin levels, including disease severity, accompanying clinical features, and treatment‐related variables [11].

## 2 Materials and Methods

### 2.1 Study Design and Participants

This prospective observational study was conducted at Fırat University Hospital and Fethi Sekin City Hospital from October 2021 to March 2022. The institutional ethics committee approved the protocol (approval number: 2021‐3769, date: 22.09.2021), and the principles of the Declaration of Helsinki were adhered to throughout. Written informed consent was obtained from all participants or their legal representatives.

Ninety adults were enrolled and classified according to the Turkish Ministry of Health COVID‐19 management guidelines [12]. The control group comprised healthy individuals with a negative SARS‐CoV‐2 RT‐PCR result within 48 h, no prior COVID‐19 history, no recent vaccination, and no respiratory symptoms. The mild COVID‐19 group (*n* = 30) included patients with a positive SARS‐CoV‐2 PCR result within 1 week who presented with mild symptoms and no radiological evidence of pneumonia. The severe COVID‐19 group (*n* = 30) comprised hospitalized patients with confirmed SARS‐CoV‐2 infection and radiologically confirmed pneumonia who were receiving the standard pulse methylprednisolone protocol in accordance with national treatment guidelines at the time of enrollment.

In all COVID‐19 groups, blood samples were collected within the first week of symptom onset. In the severe group, all patients were receiving corticosteroid therapy at the time of blood sampling; however, the exact day of sampling relative to corticosteroid initiation was not systematically recorded. Exclusion criteria included chronic salivary gland disorders, autoimmune diseases, active malignancy, recent immunosuppressive therapy, severe renal insufficiency, chronic liver disease, and pregnancy.

### 2.2 Sample Analysis

Serum samples were analyzed using commercially available ELISA kits for histatin‐1 (Catalog no: E5406Hu), histatin‐3 (Catalog no: E5407Hu), and histatin‐5 (Catalog no: E1444Hu) from Bioassay Technology Laboratory (Shanghai, China). All procedures adhered to manufacturer specifications. The histatin‐1 assay had a measurement range of 10–2000 ng/L (sensitivity: 4.39 ng/L); the histatin‐3 assay measured 0.05–20 µg/mL (sensitivity: 0.029 µg/mL); the histatin‐5 assay detected 0.5–100 ng/mL (sensitivity: 0.249 ng/mL). Intra‐assay and inter‐assay coefficients of variation were below 8% and 10%, respectively.

### 2.3 Statistical Analysis

Sample size was determined a priori: 30 participants per group provided 80% power to detect a 30% difference in histatin levels (α = 0.05). All analyses were performed using SPSS version 26.0 (IBM Corp., Armonk, NY, USA). Data distribution was assessed using Shapiro–Wilk tests. As histatin concentrations were not normally distributed (Shapiro–Wilk *p* < 0.05 for all groups), Kruskal–Wallis tests were used for overall three‐group comparisons, followed by Mann–Whitney *U* tests for pairwise analyses. Age was compared using one‐way ANOVA with Tukey's post‐hoc test; categorical variables were compared using the *χ* ^2^ test. Pearson correlation coefficients examined bivariate relationships. Multiple linear regression identified independent predictors of histatin‐3, adjusting for age, sex, disease severity, C‐reactive protein, and lymphocyte count. A two‐tailed *p* < 0.05 was considered significant.

## 3 Results

### 3.1 Baseline Characteristics

Demographic and clinical characteristics are presented in Table 1. Sex distribution was comparable across groups (*χ* ^2^ *p* = 0.823). A significant age gradient was observed (ANOVA *p* < 0.001): patients with severe COVID‐19 were the oldest (66.9 ± 10.5 years), followed by mild cases (57.0 ± 14.4 years) and controls (46.1 ± 17.2 years), with significant differences between all pairs (controls vs. mild *p* = 0.010; controls vs. severe *p* < 0.001; mild vs. severe *p* = 0.029). Chronic comorbidities were more prevalent in the severe group (63.33%) than in the mild group (43.33%). The median total illness duration was 7 days (IQR: 5–7) in the mild group and 20 days (IQR: 15–30) in the severe group (*p* < 0.001).

**Table 1:** Demographic and clinical characteristics of study participants.

Parameter	Controls (*n* = 30)	Mild COVID‐19 (*n* = 30)	Severe COVID‐19 (*n* = 30)	*p* value
Age (years)	46.1 ± 17.2	57.0 ± 14.4	66.9 ± 10.5	**< 0.001**
Gender (male/female)	13/17	16/14	15/15	0.823
Chronic disease, *n* (%)		13 (43.33%)	19 (63.33%)	
Total illness duration (days)		7 (5–7)	20 (15–30)	**< 0.001**
Hospital stay (days)		3.9 ± 3.9	22.0 ± 12.9	
ICU stay (days)		0	20.1 ± 12.8	
Mortality, *n* (%)		1 (3.33%)	26 (86.66%)	

*Note:* Data are presented as mean ± SD, median (IQR), or *n* (%). Age was compared across the three groups using one‐way ANOVA; gender was compared using the *χ* ^2^ test. Total illness duration was compared between mild and severe COVID‐19 groups using the Mann–Whitney *U* test. Pairwise post‐hoc comparisons for age are reported in the main text. Bold *p* values indicate statistical significance (*p* < 0.05).

Abbreviations: COVID‐19, coronavirus disease 2019; ICU, intensive care unit; IQR, interquartile range; SD, standard deviation.

### 3.2 Laboratory Profiles

Laboratory parameters stratified by disease severity are detailed in Table 2. Inflammatory markers increased markedly with severity: C‐reactive protein was 162.56 ± 125.11 mg/L in severe versus 29.70 ± 30.23 mg/L in mild disease (*p* < 0.001). The neutrophil‐to‐lymphocyte ratio demonstrated a nearly three‐fold increase in severe cases (11.83 ± 10.50 vs. 4.34 ± 5.06; *p* < 0.001). Serum albumin was significantly reduced in the severe group (3.22 ± 0.43 vs. 4.41 ± 0.28 g/dL; *p* < 0.001), reflecting the catabolic state of critical illness.

**Table 2:** Laboratory parameters in COVID‐19 patients.

Parameter	Mild COVID‐19 (*n* = 30)	Severe COVID‐19 (*n* = 30)	*p* value
WBC (×10^9^/L)	4.96 ± 2.20	9.47 ± 4.10	**< 0.001**
Neutrophil (×10^9^/L)	3.64 ± 2.38	8.45 ± 3.22	**< 0.001**
Lymphocyte (×10^9^/L)	1.24 ± 0.78	0.90 ± 0.42	**0.032**
Platelet (×10^9^/L)	178.17 ± 52.31	219.85 ± 86.45	0.743
Hemoglobin (g/dL)	13.88 ± 2.01	12.47 ± 2.79	0.121
CRP (mg/L)	29.70 ± 30.23	162.56 ± 125.11	**< 0.001**
Ferritin (ng/mL)	215.00 ± 162.97	598.93 ± 431.58	**0.004**
D‐dimer (mg/L)	0.81 ± 0.42	2.71 ± 2.36	0.144
LDH (U/L)	473.10 ± 134.73	618.62 ± 231.80	**0.019**
ALT (U/L)	26.23 ± 13.28	73.30 ± 128.77	**0.003**
AST (U/L)	33.44 ± 11.56	116.60 ± 234.00	**< 0.001**
Albumin (g/dL)	4.41 ± 0.28	3.22 ± 0.43	**< 0.001**
Creatinine (mg/dL)	0.99 ± 0.21	1.30 ± 1.64	0.869
NLR	4.34 ± 5.06	11.83 ± 10.50	**< 0.001**

*Note:* Data presented as mean ± SD. Mann–Whitney *U* test used for all comparisons. Bold *p* values indicate statistical significance (*p* < 0.05).

Abbreviations: ALT, alanine aminotransferase; AST, aspartate aminotransferase; CRP, C‐reactive protein; LDH, lactate dehydrogenase; NLR, neutrophil‐to‐lymphocyte ratio; WBC, white blood cell count.

### 3.3 Serum Histatin Concentrations Across Disease Severity

**Figure 1:**
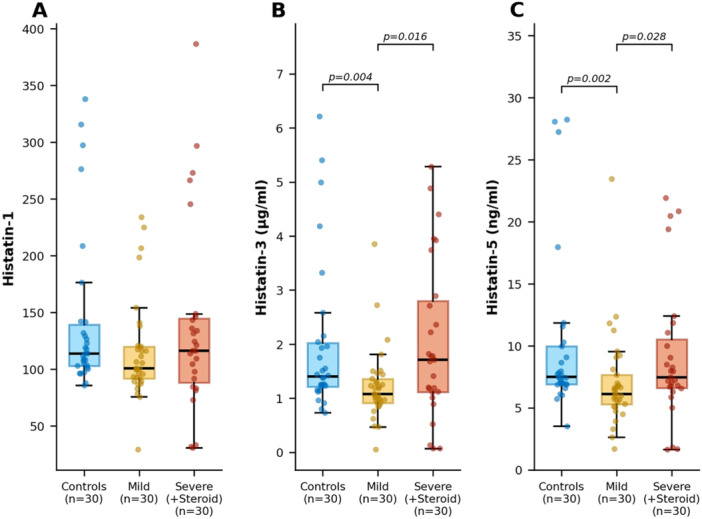
Serum histatin concentrations according to disease category. Box‐and‐whisker plots with individual data points show serum levels of (A) histatin‐1, (B) histatin‐3, and (C) histatin‐5 in healthy controls, patients with mild COVID‐19, and patients with severe COVID‐19 receiving corticosteroid therapy. Boxes represent the interquartile range, center lines indicate the median, and whiskers indicate the minimum and maximum values. Exact *p* values for significant pairwise comparisons are shown above the relevant groups.

Serum histatin concentrations are presented in Table 3 and Figure 1. Although the pairwise comparison between controls and mild COVID‐19 reached nominal significance, histatin‐1 was not interpreted as significantly different across groups because the overall Kruskal–Wallis test was not significant (Kruskal–Wallis *p* = 0.161). In contrast, histatin‐3 showed significant inter‐group variation (Kruskal–Wallis *p* = 0.007). Patients with mild COVID‐19 without pneumonia exhibited a 38% reduction compared with controls (1.22 ± 0.66 vs. 1.98 ± 1.39 µg/mL; *p* = 0.004). Unexpectedly, patients with severe COVID‐19 with pneumonia, all of whom were receiving corticosteroid therapy, did not exhibit a further decline; their histatin‐3 levels were comparable to controls (2.04 ± 1.44 µg/mL; *p* = 0.987 vs. controls) and significantly higher than the mild group (*p* = 0.016).

**Table 3:** Serum histatin concentrations by disease category.

Parameter	Controls (*n* = 30)	Mild COVID‐19 (*n* = 30)	Severe COVID‐19 (*n* = 30)	*p* (K–W)	*p* ctrl vs. mild	*p* mild vs. severe
Histatin‐1(ng/L)	142.66 ± 69.39 (113.74)	115.61 ± 43.33 (100.57)	137.70 ± 83.57 (116.22)	0.161	**0.040**	0.309
Histatin‐3 (µg/mL)	1.98 ± 1.39 (1.40)	1.22 ± 0.66 (1.08)	2.04 ± 1.44 (1.71)	**0.007**	**0.004**	**0.016**
Histatin‐5 (ng/mL)	10.09 ± 6.53 (7.49)	7.02 ± 3.74 (6.12)	9.17 ± 5.44 (7.46)	**0.006**	**0.002**	**0.028**

*Note:* Data presented as mean ± SD (median). Histatin data were not normally distributed (Shapiro–Wilk *p* < 0.05 for all groups); Kruskal–Wallis (K–W) test used for overall comparison, Mann–Whitney *U* test for pairwise comparisons. Bold *p* values indicate statistical significance (*p* < 0.05).

Abbreviations: ctrl, controls; K–W, Kruskal–Wallis; sev, severe COVID‐19.

Histatin‐5 demonstrated a parallel pattern (Kruskal–Wallis *p* = 0.006). Mild cases showed a 30% reduction relative to controls (7.02 ± 3.74 vs. 10.09 ± 6.53 ng/mL; *p* = 0.002), while levels in the severe group were comparable to controls (9.17 ± 5.44 ng/mL; *p* = 0.700) and significantly higher than in mild disease (*p* = 0.028). The concordance of this pattern across both isoforms provides convergent evidence that circulating histatins are dynamically modulated across the COVID‐19 severity spectrum.

### 3.4 Correlation Analyses

**Figure 2:**
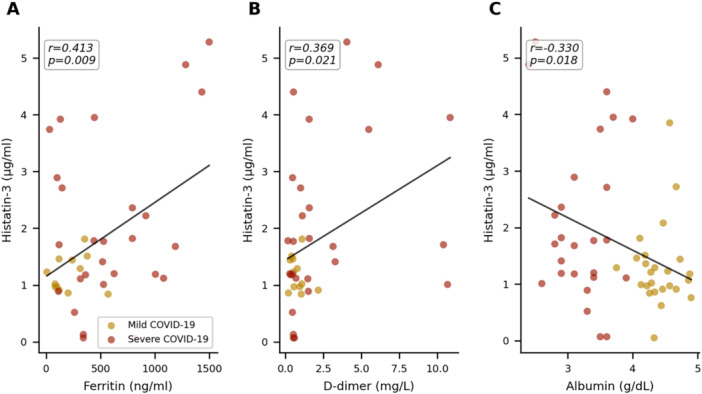
Correlation plots of histatin‐3 with selected laboratory parameters in patients with COVID‐19. Scatter plots show the associations between serum histatin‐3 levels and (A) ferritin, (B) d‐dimer, and (C) albumin in patients with COVID‐19 (mild and severe groups combined). Lines indicate linear trend lines. Pearson correlation coefficients (*r*) and corresponding *p* values are displayed within each panel. For figure economy, only the most clinically informative significant correlations of histatin‐3 are displayed in Figure 2, whereas the complete correlation results, including neutrophil count, are presented in Table 4.

Correlation analyses among COVID‐19 patients are summarized in Table 4 and Figure 2. Histatin‐3 demonstrated significant positive correlations with ferritin (*r* = 0.413; *p* = 0.009), D‐dimer (*r* = 0.369; *p* = 0.021), and neutrophil count (*r* = 0.300; *p* = 0.024), as well as a significant inverse correlation with albumin (*r *= −0.330; *p* = 0.018). The strongest association was with ferritin, suggesting a link between histatin‐3 and the acute‐phase response. Histatin‐5 did not exhibit significant correlations with any of the examined laboratory parameters.

**Table 4:** Correlations of histatin isoforms with laboratory parameters in COVID‐19 patients.

Parameter	Histatin‐3 r (*p* value)	Histatin‐5 r (*p* value)
Neutrophil count	**0.300 (0.024)**	0.017 (0.903)
CRP	0.058 (0.674)	0.077 (0.576)
Ferritin	**0.413 (0.009)**	0.195 (0.234)
d‐dimer	**0.369 (0.021)**	0.129 (0.434)
LDH	0.081 (0.588)	0.042 (0.777)
Albumin	**0.330 (0.018)**	0.182 (0.202)

*Note:* Pearson correlation coefficients calculated among COVID‐19 patients (mild and severe groups combined). Bold values indicate statistical significance (*p* < 0.05).

Abbreviations: CRP, C‐reactive protein; LDH, lactate dehydrogenase.

### 3.5 Clinical Outcomes

Clinical outcome data are presented in Table 1. The mean hospital stay was 22.0 ± 12.9 days, with all severe patients requiring intensive care (mean ICU stay: 20.1 ± 12.8 days). Twenty‐six of 30 patients with severe disease died (86.66%), reflecting the critically ill nature of this population during the pre‐vaccination pandemic period.

Comparison of survivors (*n *= 4) and non‐survivors (*n *= 26) in the severe group (Table 5) revealed numerically higher histatin‐3 among survivors (3.26 ± 2.12 vs. 1.83 ± 1.28 µg/mL; *p* = 0.183) and histatin‐5 (11.93 ± 6.01 vs. 8.69 ± 5.46 ng/mL; *p* = 0.168). Although these differences did not attain statistical significance owing to the small survivor sample, survivors exhibited significantly higher ferritin (1244.75 ± 205.59 vs. 486.61 ± 354.03 ng/mL; *p* = 0.005).

**Table 5:** Comparison of survivors and non‐survivors in severe COVID‐19.

Parameter	Survivors (*n* = 4)	Non‐survivors (*n* = 26)	*p* value
Age (years)	68.25 ± 4.79	66.70 ± 11.22	0.918
Histatin‐1 (ng/L)	142.11 ± 69.25	136.93 ± 88.95	0.974
Histatin‐3 (µg/mL)	3.26 ± 2.12	1.83 ± 1.28	0.183
Histatin‐5 (ng/mL)	11.93 ± 6.01	8.69 ± 5.46	0.168
WBC (×10^9^/L)	12.47 ± 7.19	9.19 ± 3.70	0.473
Lymphocyte (×10^9^/L)	1.04 ± 0.59	0.89 ± 0.51	0.220
CRP (mg/L)	241.88 ± 150.45	148.76 ± 118.61	0.232
Ferritin (ng/mL)	1244.75 ± 205.59	486.61 ± 354.03	**0.005**
d‐dimer (mg/L)	3.44 ± 2.34	2.42 ± 3.45	0.339
LDH (U/L)	819.25 ± 351.04	582.14 ± 193.13	0.189
Albumin (g/dL)	2.73 ± 0.33	3.31 ± 0.38	**0.022**

*Note:* Data presented as mean ± SD. Mann–Whitney *U* test used for all comparisons. Bold *p* values indicate statistical significance (*p* < 0.05).

Abbreviations: CRP, C‐reactive protein; LDH, lactate dehydrogenase; WBC, white blood cell count.

### 3.6 Multivariable Analysis

In multiple linear regression among COVID‐19 patients—adjusted for age, sex, disease severity, CRP, and lymphocyte count—severe disease status emerged as the strongest independent predictor of histatin‐3 (*β* = 1.02; 95% CI: 0.28–1.76; *p* = 0.008), indicating that patients with pneumonia receiving corticosteroids had significantly higher histatin‐3 levels than those with mild disease after controlling for confounders. CRP was independently and inversely associated with histatin‐3 (*β* = −0.004; 95% CI: −0.007 to −0.000; *p* = 0.034), suggesting that systemic inflammation suppresses histatin‐3 production. Lymphocyte count was not an independent predictor (*β* = 0.109; *p* = 0.687).

## 4 Discussion

This study provides the first characterization of circulating histatin levels across the clinical spectrum of COVID‐19, from mild disease without pneumonia to severe pneumonia requiring intensive care. The principal finding is that serum histatin‐3 and histatin‐5 are significantly reduced in patients with mild COVID‐19, consistent with the hypothesis that SARS‐CoV‐2 infection suppresses histatin production. However, a striking and unexpected observation was that patients with severe COVID‐19 with pneumonia did not exhibit further histatin reductions; instead, their levels were comparable to those of healthy controls.

The reduction in histatin‐3 and histatin‐5 during mild COVID‐19 likely reflects the convergence of several mechanisms. Mild COVID‐19 predominantly involves the upper respiratory tract, where the salivary glands, the primary source of histatins, are directly exposed to viral injury. SARS‐CoV‐2 has been shown to infect salivary gland acinar cells via ACE2 receptors [13, 14], causing cellular dysfunction and impaired secretory function [8]. Additionally, the pro‐inflammatory milieu of acute infection, driven by cytokines such as interleukin‐6 and tumor necrosis factor‐α, suppresses antimicrobial peptide production [15], and accelerated consumption during the immune response may further deplete reserves [16].

The unexpected preservation of histatin levels in the severe group warrants careful interpretation. Several non‐mutually exclusive explanations may account for this finding. First, severe COVID‐19 predominantly involves the lower respiratory tract, and salivary glands may be relatively less affected compared with mild disease, in which upper airway tropism is dominant. Second, all patients in the severe group were receiving pulse corticosteroid therapy at the time of sampling. Glucocorticoids are known to modulate antimicrobial peptide production through multiple mechanisms: reduction of cytokine‐mediated transcriptional suppression [17], direct cytoprotective effects on glandular epithelium, and restoration of secretory pathways. Third, severe systemic inflammation may be associated with compensatory changes in histatin levels, potentially involving secondary tissue sources such as the respiratory epithelium and neutrophils [18].

It must be emphasized that this study was not designed to evaluate the effect of corticosteroids on histatin levels. The observed preservation of histatins in the severe group is a cross‐sectional observation, and pre‐treatment histatin levels were not available. It is therefore not possible to determine whether corticosteroids restored previously suppressed histatin levels, prevented their decline, or whether the finding is entirely attributable to the differential anatomical involvement of mild versus severe disease. Disentangling these contributions would require a longitudinal design with serial sampling before and after corticosteroid initiation, ideally including a severe COVID‐19 comparator group not receiving corticosteroids.

The significant correlations of histatin‐3 with ferritin (*r* = 0.413; *p* = 0.009), D‐dimer (*r* = 0.369; *p* = 0.021), and albumin (*r* = −0.330; *p* = 0.018) represent notable findings. The positive association with ferritin—an acute‐phase reactant markedly elevated in severe COVID‐19—suggests that histatin‐3 may participate in a coordinated acute‐phase response involving both antimicrobial peptides and iron metabolism pathways. The inverse correlation with albumin is consistent with the well‐established role of hypoalbuminemia as a marker of disease severity in COVID‐19. The additional correlation with neutrophil count (*r* = 0.300; *p* = 0.024) suggests that histatin‐3 may be associated with inflammatory and immune‐related changes in COVID‐19. Notably, histatin‐5 did not show significant correlations with these parameters, positioning histatin‐3 as the more informative isoform. However, these associations are correlational, and this study did not include functional assays to evaluate the biological activity of circulating histatins.

The mortality rate in the severe group (86.66%) was high, reflecting the critically ill, pre‐vaccination population studied. Although histatin differences between survivors and non‐survivors did not reach significance, the consistent trend of higher levels among survivors, together with the significant ferritin difference (*p* = 0.005), suggests a possible prognostic dimension warranting investigation in larger cohorts.

Several limitations should be acknowledged. First, the cross‐sectional design precludes causal inferences and longitudinal tracking. Second, the absence of a severe COVID‐19 group not receiving corticosteroids prevents definitive attribution of preserved histatin levels to treatment versus disease characteristics. Therefore, the observed pattern should be interpreted as a cross‐sectional association rather than evidence of treatment effect. Third, the differential anatomical involvement (upper vs. lower airway) between mild and severe disease is a potential confounder. Fourth, age differences, although statistically adjusted, may partially confound results. Fifth, the high mortality and small survivor sample (*n* = 4) limit prognostic analyses. Additional limitations include single‐center recruitment, lack of viral variant data, and absence of functional assays.

## 5 Conclusions

This study demonstrates that serum histatin‐3 and histatin‐5 are significantly reduced in patients with mild COVID‐19 without pneumonia, while levels in patients with severe COVID‐19 with pneumonia, who were concurrently receiving corticosteroid therapy, are comparable to those of healthy controls. Whether the preservation of histatin levels in severe disease reflects the influence of corticosteroid treatment, the differential anatomical distribution of disease, or both, cannot be determined from this cross‐sectional design. The significant correlations of histatin‐3 with ferritin, D‐dimer, and albumin suggest that histatin‐3 may be linked to acute‐phase and inflammatory responses during COVID‐19. Future longitudinal studies with serial sampling and inclusion of untreated severe comparator groups are needed to clarify the respective contributions of disease severity and corticosteroid therapy to circulating histatin dynamics.

## Author Contributions

**Umut Aydın:** conceptualization, methodology, investigation, data curation, formal analysis, writing – original draft, writing – review and editing, supervision. **Deccane Düzenci:** investigation, resources, data curation, validation. **Ramazan Fazıl Akkoç:** methodology, formal analysis, visualization, and validation. **Ahmet Karataş:** investigation, resources, writing – review and editing.

## Funding

The authors have nothing to report.

## Conflicts of Interest

The authors declare no conflicts of interest.

## Data Availability

Anonymized data sets are available from the corresponding author upon reasonable request, subject to ethical approval and data protection requirements.
